# Methazolamide Can Treat Atherosclerosis by Increasing Immunosuppressive Cells and Decreasing Expressions of Genes Related to Proinflammation, Calcification, and Tissue Remodeling

**DOI:** 10.1155/2024/5009637

**Published:** 2024-07-23

**Authors:** Hongji Zhou, Rui Zhang, Min Li, Fuyan Wang, Yuxia Gao, Kehua Fang, Jinbao Zong, Xiaotian Chang

**Affiliations:** ^1^ Medical Research Center The Affiliated Hospital of Qingdao University, Wutaishan Road 1677, Qingdao 266000, China; ^2^ Department of Cardiology The Affiliated Hospital of Qingdao University, Wutaishan Road 1677, Qingdao 266000, China; ^3^ Clinical Laboratory and Central Laboratory Qingdao Hiser Hospital Affiliated of Qingdao University (Qingdao Traditional Chinese Medicine Hospital), Road Renmin 4, Qingdao 266033, Shandong Province, China; ^4^ Shandong Engineering Research Center of Bacterial Anti-tumor Drugs and Cell Therapy, Jingshi Road 7000, Jinan 250000, Shandong Province, China; ^5^ Clinical Laboratory The Affiliated Hospital of Qingdao University, Wutaishan Road 1677, Qingdao, Shandong 266000, China

## Abstract

It has been reported that carbonic anhydrase I (CA1) is a target for the diagnosis and therapy of atherosclerosis (AS) since CA1 can promote AS aortic calcification. We also found that methazolamide (MTZ), a drug for glaucoma treatment and an inhibitor of carbonic anhydrases, can treat AS by inhibiting calcification in aortic tissues. This study focused on the therapeutic mechanism of MTZ and the pathogenic mechanism of AS. In this study, a routine AS animal model was established in ApoE−/− mice, which were treated with MTZ. The aortic tissues were analyzed using single-cell sequencing. MTZ significantly increased the proportions of B-1/MZB B cells with high expressions of Nr4A1 and Ccr7, CD8+CD122+ Treg-like cells with high Nr4A1 expression, and smooth muscle cells with high Tpm2 expression. These cells or their marker genes were reported to exert immunosuppressive, anti-proinflammatory, and atheroprotective effects. MTZ also decreased the proportions of endothelial cells with high expressions of Retn, Apoc1, Lcn2, Mt1, Serpina3, Lpl, and Lgals3; nonclassical CD14+CD16++ monocytes with high expressions of Mt1, Tyrobp, Lgals3, and Cxcl2; and Spp1+ macrophages with high expressions of Mmp-12, Trem2, Mt1, Lgals3, Cxcl2, and Lpl. These cells or their marker genes have been reported to promote inflammation, calcification, tissue remodeling, and atherogenesis. A significant decrease in the proportion of CD8+CD183 (CXCR3)+ T cells, the counterpart of murine CD8+CD122+ T cells, was detected in the peripheral blood of newly diagnosed AS patients rather than in that of patients receiving anti-AS treatments. These results suggest that MTZ can treat AS by increasing immunosuppressive cells and decreasing expressions of genes related to inflammation, calcification, and tissue remodeling.

## 1. Introduction

The degree of aortic calcification is strongly correlated with atherosclerosis (AS) severity and accelerates AS progression [1]. The determining step leading to AS calcification is calcium precipitation, which traps cholesterol in the aortic plaque [2]. Carbonic anhydrases (CAs) catalyze carbon dioxide into bicarbonate, which easily combines with calcium ions to form calcium carbonate, which sequentially accelerates calcium phosphate mineralization under physiological and pathological conditions [3]. In 2013, Ando et al. [4] detected the autoantigenicity of carbonic anhydrase 1 (CA1), a CA family member, in tissues of abdominal aortic aneurysms that often occur in AS patients. We previously reported that CA1 is highly expressed in human AS aortic tissues and in vascular smooth muscle cells (VSMCs) with *β*-glycerophosphate-induced calcification. Treatment with an anti-CA1 siRNA and methazolamide (MTZ), a CA inhibitor and a drug for glaucoma treatment, significantly suppressed calcification in cultured VSMCs. Furthermore, treatment of an AS mouse model with MTZ significantly alleviated AS symptoms and plaque formation, and CA1 expression in aortic tissues and proinflammatory cytokine secretion in peripheral blood simultaneously decreased [5]. We recently found that M1 macrophages, which are usually highly abundant in AS aortic tissues, can promote atherosclerotic calcification by producing TNF-*α* to stimulate CA1 and CA2 expression in VSMCs [6]. Additionally, a strong relationship between AS and glaucoma in patients was demonstrated due to the expression of CA or CA1 in both AS aortic tissues and glaucoma aqueous humor, and AS patients with glaucoma simultaneously had reduced low-density lipoprotein (LDL) levels, a biochemical index of AS, when they were treated with MTZ to cure glaucoma, which supports that MTZ can treat AS [7]. The above results demonstrate that CA1 can promote calcification and subsequently stimulate AS pathogenesis and that MTZ can treat AS by inhibiting CA1-mediated calcification.

In recent years, the relationship between CA1 and AS has attracted increased amounts of attention. Thirty-five cardiac drugs, especially those drugs that are frequently used for AS treatment [8], significantly inhibit CA1 and CA2 activity. By screening many circulating proteins with potential risk prediction for cardiovascular events, CA1 was confirmed to be the most predictive for subclinical carotid AS [9]. Our and other studies supported that CA1 is involved in AS pathogenesis. However, the functional mechanism of MTZ in the treatment of AS has not been fully elucidated.

In the present study, an AS mouse model was established by administering a high-fat diet to ApoE−/− mice, which were treated or preventively treated with MTZ as we previously described [5]. Single-cell sequencing was applied to analyze the mouse aortas. The analytical results were verified with peripheral blood samples from AS patients. This study aimed to elucidate the mechanism by which MTZ exerts therapeutic effects on AS. Another purpose of this study was to further explore the molecular and cellular mechanism of AS with MTZ as an active intervention drug to suppress aortic calcification.

## 2. Materials and Methods

### 2.1. Establishment of an AS Mouse Model

Eight-week-old healthy male C57BL/6J ApoE−/− mice were randomly divided into four groups with 18 mice in each group. The healthy control group was fed a normal diet, while the other three groups were fed a high-fat diet (1% cholesterol and 10% fat) for a total of 24 weeks. Healthy controls were given normal saline. The AS model group was treated with normal saline. The MTZ-treated group was treated with normal saline for the first 1–12 weeks and with MTZ for 13–24 weeks. The MTZ-preventive treated group was treated with MTZ for 24 weeks. Normal saline was intragastrically administered at a dose of 0.1 mL/10 g, and MTZ dissolved in normal saline was intragastrically administered at a dose of 25 mg/kg. These animals were treated once every other day. MTZ was obtained from Hangzhou Aoyipollen Pharmaceutical (China). The dose used was based on previous experimental results [5]. All animals were housed under specific pathogen-free conditions and handled in accordance with the guidelines of the Declaration of Helsinki. The experimental design was approved by the Ethics Committee of the Affiliated Hospital of Qingdao University (QYFY WZLL 27438).

### 2.2. Hematoxylin and Eosin (H&E) Staining

Mouse aorta tissues were collected from those ApoE−/− mice with AS or not and fixed with 4% paraformaldehyde. The tissues were then embedded in paraffin following a series of ethanol dehydrations via a routine protocol. They were sectioned by a microtome and hydrated by a series of ethanol solutions. The tissue sections were stained with HE staining solution and dehydrated with ethanol in a routine manner. The tissue structure was examined via microscopy.

### 2.3. Sudan IV Staining

Fresh aortic tissues from the mice were sliced at low temperature by a freezing microtome. The frozen sections were placed on glass slides and slightly soaked in 70% ethanol. They were then immersed in Sudan IV dye solution for 1–2 min and washed with 70% ethanol to wash away excess dye solution. The tissue structure was examined via microscopy.

### 2.4. Biochemical Examination

Mouse peripheral blood was obtained in a heparin blood collection tube. The supernatant was collected by centrifugation and analyzed using an automatic biochemical analyzer (Mindray, China). The levels of apolipoprotein (AI), high-density lipoprotein (HDL), LDL, nitric oxide (NO), and triglyceride (TG) in the mouse plasma were measured.

### 2.5. Routine Mouse Blood Test

Mouse peripheral blood was collected in an EDTA blood collection tube and analyzed with an automatic hematology analyzer (Mindray). The levels of eosinophils (EOS), lymphocytes (Lym), monocytes (Mon), neutrophils (Neu), and white blood cells (WBCs) were counted.

### 2.6. Measurement of Serum Cytokine Levels

Mouse peripheral blood was collected, and the supernatant was obtained by centrifugation. The levels of cytokines in the blood samples were measured using a Mouse Inflammation Panel (13-plex) (BioLegend, USA) according to the manufacturer's instructions. Standard or test samples were mixted with magnetic beads and assay buffer, and the Mouse Inflammation Panel Detection solution as well as various anticytokine antibodies and streptavidin-PE was then added. The cytokine levels were determined using a flow cytometry (Apogee A50 instrument, NovoCyte D2040R, UK). This protocol was applied in our previous study [10].

### 2.7. Evaluation of Lymphocyte Subtypes

Mouse peripheral blood was obtained and collected into an EDTA blood collection tube. The red bloods in the samples were lysed with red blood cell lysis buffer (Solarbio, China). CD3+ CD19− T cells and CD3− CD19+ B cells were identified with an APC-conjugated antimouse CD3e antibody (eBioscience, USA), CD3− CD19+ B cells were identified with phycoerythrin (PE)-conjugated antimouse CD19 antibody (eBioscience), and NK cells were identified with an APC-conjugated antimouse CD3e antibody and PE-conjugated antimouse NK1.1 antibody (BioLegend, USA). The lymphocyte subtypes were classified by a flow cytometer (Agilent, USA). The proportions of each cell subtypes were determined by calculating the proportion of that cell type within the total lymphocyte population. This protocol was applied in our previous study [10].

### 2.8. Measurement of Treg Cell Proportions Using Flow Cytometry

APC-conjugated antimouse CD4 and FITC-conjugated antirat CD25 antibodies (BioLegend) were added to the blood samples. After centrifugation, the pelleted cells were treated with fixation buffer (BioLegend). Following centrifugation, the fixed cells were resuspended in permeabilization wash buffer (BioLegend), and a PE-conjugated antimouse FOXP3 antibody (BioLegend) was added. Treg cell levels were measured using flow cytometry.

### 2.9. Single-Cell RNA Sequencing and Data Analysis

Aortic dissection tissues were collected from experimental mice in each group. Six aorta samples from each group were pooled together as one sample. The cells were lysed in the solution, and their mRNA was exposed to the barcoded gel beads. The barcoded mRNA was reverse-transcribed to full-length cDNA and amplified by PCR. The amplified cDNA was fragmented, end-repaired, end-tailed, subjected to index adaptor ligation, and then subjected to library amplification. The genomic platform was used to construct a 10x barcoded library by mixing the cells with 10x barcoded gel beads using microfluidic technology. The constructed libraries were subsequently sequenced using an Illumina NovaSeq 6000 system. Transcript sequences in combination with unique molecular identifiers (UMI) (a sequence consisting of 4–10 random nucleotides) and barcodes were used to determine the absolute number of each transcript molecule within a single cell. Cells with a gene number greater than 200, a UMI greater than 1,000, a log10GenesPerUMI greater than 0.7, a mitochondrial UMI less than 10%, and a red blood cell gene ratio less than 5% were considered high-quality cells, and double-cell removal and downstream analysis were subsequently performed using DoubletFinder software.

Cells with similar expression profiles can be clustered together to form a cell population. The mutual nearest neighbors (MNN) dimensionality reduction algorithm was used to eliminate batch effects, and then single-cell clustering was visualized based on the dimensionality reduction results of the MNN and UMAP (Unified Manifold Approximation and Projection) algorithms.

The Presto test was used to determine the difference between the specified cell population and all other cell populations. A gene was considered a specific marker gene for each cell population if it had higher expression in more than 25% of the cluster than in other clusters.

The SingleR package was used to identify cell type annotations based on the ImmGen reference dataset. Unidentified cells were annotated according to the most correlated cell type in the reference dataset.

Differentially expressed genes (DEGs) were screened using the FindMarkers tool of the Seurat package. DEGs were further analyzed according to their fold change and *p* value. The criteria were as follows: *p*  < 0.01 and log2FoldChange ≥ 1.5 or ≤ −1.5. After screening the core genes, Gene Ontology (GO) and Kyoto Encyclopedia of Genes and Genomes (KEGG) enrichment analyses were performed to determine the activation of pathways in the samples.

This study not only compared normal mice and AS mice but also compared AS mice with MTZ-treated AS mice and MTZ-preventive treated AS mice. In this way, we can accurately identify the genes and pathways related to AS and MTZ treatment by narrowing the screening range.

### 2.10. Measurement of CD8+CD183 (CXCR3)+ Cell Levels Using Flow Cytometry

Blood samples from patients with AS or healthy subjects were collected at the Qingdao Hiser Hospital Affiliated with Qingdao University in Qingdao (Shandong, China) from March 2023 to May 2023. Twenty-nine patients with AS were selected. These patients were newly diagnosed (*n* = 13) or treated (*n* = 15) with various medicines. AS patients with cardiomyopathy, liver or kidney dysfunction, infection, cancer, autoimmune disease, or sepsis were excluded from the study. The healthy control subjects consisted of 28 healthy people who showed no clinically obvious coronary artery abnormalities. Written informed consent was obtained from all patients and healthy volunteers. The details of all the samples are provided in Supplementary file 1. The experimental design was approved by the Ethics Committee of the Affiliated Hospital of Qingdao University (QYFY WZLL 27438).

Anti-CD3 (FITC) (Biosciences, USA), anti-CD4 (pc7) (Biosciences), anti-CD8 (APCCY7) (Biosciences), anti-CD45 (Percpcy5.5) (Biosciences), and anti-CD183 (APC) (Biosciences) antibodies were used to detect CD8+CD183 (CXCR3)+ cells in the blood samples using flow cytometry by a routine way.

### 2.11. Statistical Analysis

The blood biochemistry, routine blood test, lymphocyte, cytokine, and flow cytometry data were analyzed using GraphPad Prism 8.0. If the data were consistent with homogeneity of variance and had a normal distribution, one-way ANOVA was used to test for significant differences among multiple groups, and the LSD *t*-test was used for comparisons between two groups. If the data did not fit a normal distribution or homogeneity of variance, the Kruskal‒Wallis test was applied to evaluate significant differences among multiple groups. If *p* value was less then 0.05, the differences were considered statistically significant.

## 3. Results

### 3.1. MTZ Showed Therapeutic Effects on Mouse AS

**Figure 1:**
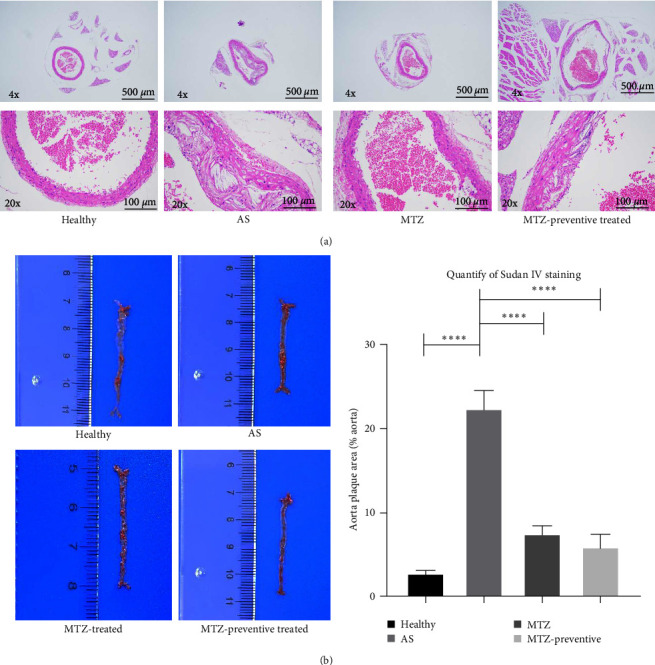
Hematoxylin and eosin (H&E) staining and Sudan IV staining of mouse aortic tissues. (a) Aortic tissues of the experimental mice were analyzed with H&E. (b) Aortic tissues from the experimental mice were stained with Sudan IV, and the signals were quantified. Aortic plaques were increased in AS aortic tissues and decreased in aortic tissues from MTZ-treated and MTZ-preventively treated mice.  ^*∗∗∗∗*^ represents a *p* value less than 0.0001.

ApoE−/− mice were fed a high-fat diet for 24 weeks. The aorta tissues were collected and examined. HE staining revealed that the aortic wall structures of the healthy mice were well developed and that the inner wall was very smooth. Compared with those of the aortic wall of healthy mice, the aortic wall of AS model mice was significantly thickened, and numerous AS plaques and massive foam cells were present. Moreover, the smooth muscle layer of aortic tissues is loosened and structurally disturbed, and typical AS plaques protrude from the intima. Sudan IV staining also revealed numerous AS plaques in the aortas of AS model mice. The above observations demonstrated successful establishment of the AS mouse model. Compared to those of the aortic wall of AS model mice, the aortic walls of MTZ-treated mice and mice that received MTZ preventive treatment exhibited reduced AS pathology, and the volume and number of AS plaques in the aorta decreased following MTZ treatment. Quantitative analysis via Sudan IV staining also revealed increased aortic plaque density in AS aortic tissues and decreased staining density in aortic tissues from treated mice (Figure 1). The above observation qualitatively and quantitatively confirmed that the administration of MTZ to ApoE−/− AS model mice significantly improved AS pathogenesis, which confirmed the previous observation of ours [5].

Compared to the healthy controls, the AS group displayed elevated levels of TG, LDL, and AI in their peripheral blood, and the MTZ treatment and MTZ-preventive treatment groups exhibited decreased levels of TG, LDL, and AI. Moreover, the levels of NO and HDL were decreased in AS model mice and increased in AS model mice that received MTZ treatment or MTZ-preventive treatment (Supplementary file 2). Additionally, compared with healthy mice, AS model mice exhibited decreases in WBC and Lym counts in their peripheral blood, and Mon and Neu counts increased. Mon and Neu levels were decreased in the MTZ-treated group and the MTZ-preventive treated group compared with those in the AS model group (Supplementary file 3). AS model mice showed high levels of IFN-*γ*, IL-1*β*, IL-6, and TNF-*α* in their peripheral blood, and MTZ-treated and MTZ-preventive treated mice showed decreased levels of IL-6, IFN-*γ*, IL-1*β*, and TNF-*α* (Supplementary file 4). Compared to the healthy controls, the AS group had decreased proportions of Treg cells in total lymphocytes in their peripheral blood, and the MTZ treatment and MTZ-preventive treatment groups had elevated proportions of Treg cells. No significant changes in other immune cell subtypes, including T cells, B cells, and NK cells, were detected among these groups (Supplementary file 5). The above observations indicated that the biochemistry, immunity, and hemogram of AS model mice were obviously altered, and these indexes were markedly restored after MTZ treatment.

### 3.2. MTZ Elevated the Number of Immunosuppressive Cells and Altered the Expression of Genes Related to Calcification, Inflammation, and AS Progress

**Figure 2:**
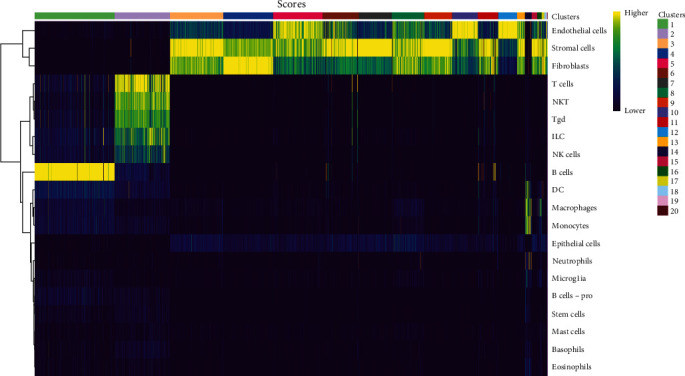
Association of cell clusters with cell types based on the Immunological Genome Project mouse database. Each row represents the annotated cell types in the reference dataset, and each column represents the unknown cell types. Yellow represents a greater correlation value, indicating that the unknown cell type most closely resembles that cell type in the reference data.

**Figure 3:**
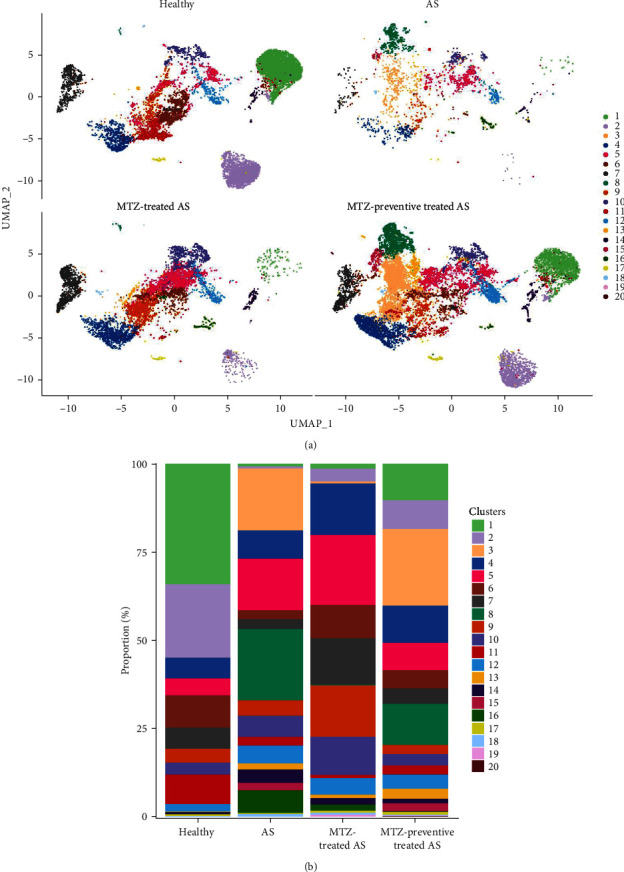
Distribution of cell types in mouse aorta tissues. (a) Diagram of the reduced dimensional clustering analysis of the four groups of mouse aorta samples. The abscissa and ordinate represent the first and second principal components of dimensionality reduction, respectively, and different cell types are distinguished by different colors. (b) Histogram of the proportion of each cell type in the four groups of tissue samples. The abscissa indicates different samples, and the ordinate indicates the proportion of the cell types in the samples.

Mouse aorta samples from the four groups of mice were analyzed using single-cell transcriptome sequencing. The number of high-quality cells was within the range of 6,908–23,963 in each sample. The average UMI number was within the range of 1,703–2,685 in each cell, and the mean number of genes per cell was 809–1,060. The raw data were submitted to the GEO repository and were approved with accession number GSE254708. These identified cells were clustered into 20 cell subsets based on expression profile similarity, and the cell numbers were within the range of 2,173–14,697 for each cell cluster (Supplementary file 6). The cell clusters were annotated as B cells, T cells, endothelial cells, fibroblasts, smooth muscle cells, myeloid cells, and neural cells (Figure 2 and Supplementary file 7). Among these 20 cell clusters, clusters 1–16 with an optimal cell number greater than 300 were ultimately selected for in-depth analysis. The proportions of clusters 1, 2, and 7 were significantly increased in the aorta samples from healthy mice, decreased in the aorta samples from AS model mice, and increased again in the aorta samples from mice treated with MTZ or pretreated with MTZ, indicating that these cell subsets are associated with human health and may play atheroprotective roles. The proportions of clusters 8, 14, and 16 were significantly increased in the AS mouse samples and decreased in the healthy control, MTZ-treated, and MTZ-preventive treated mouse samples, indicating that these cell subsets are associated with AS and may play proatherogenic roles. The proportions of clusters 3, 9, 10, 11, and 12 did not significantly change with AS progression or MTZ treatment, indicating that these cell subsets are not involved in AS pathogenesis or MTZ treatment (Figure 3). These clusters were not further analyzed in this study.

**Figure 4:**
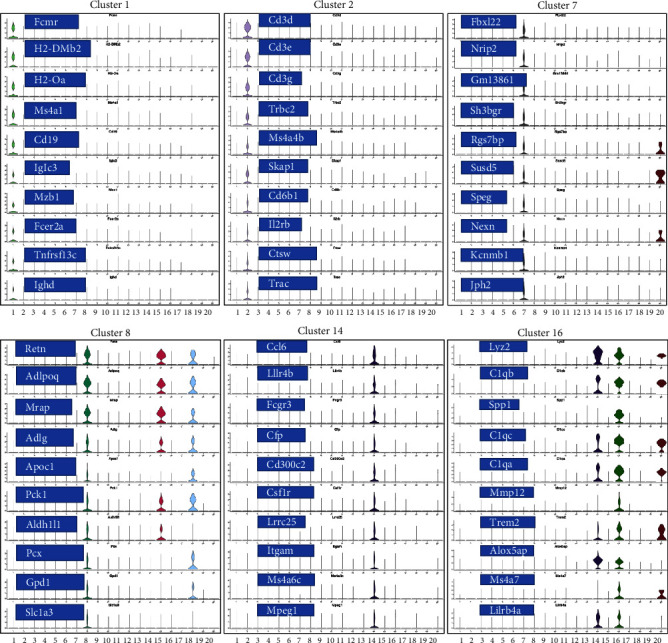
Violin diagrams for the expression of the top 10 gene markers in cell cluster 1, 2, 7, 8, 14, and 16. The abscissa is the cell cluster number, and the ordinate is the normalized gene expression.

**Figure 5:**
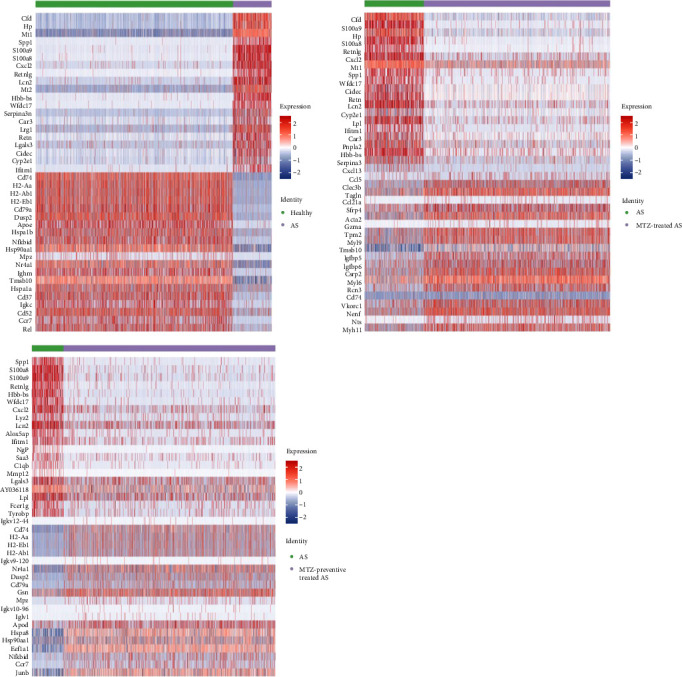
Heatmap of the top 20 differentially expressed genes (DEGs). The abscissa shows the differential sample groups, and the ordinate shows the up- and downregulation of the top 20 genes. Red indicates high gene expression, and blue indicates low gene expression.

The top 10 marker genes of clusters 1, 2, 7, 8, 14, and 16 were determined based on their expression levels in these clusters (Figure 4). DEGs were identified by comparing expression profiles between normal control mice and AS model mice, between AS model mice and AS model mice treated with MTZ, and between AS model mice and AS model mice treated with MTZ. The genes that were both among the top 20 DEGs and cluster marker genes (Supplementary file 8 and GSE254708) were emphatically analyzed in the present study. Compared with the expression profiles of normal mice, those of MTZ-treated mice and MTZ-preventive treated mice, Spp1 (secreted phosphoprotein 1 and osteopontin (OPN)), S100a9 (S100 calcium binding protein A9), S100a8 (S100 calcium binding protein A9), Cxcl2 (C-X-C motif chemokine ligand 2), Lcn2 (lipocalin 2), Hbb-bs (hemoglobin subunit beta), Wfdc17, Ifitm1 (interferon-induced transmembrane protein 1, CD225), Mt1 (metallothionein 1A), and Retnlg (resistin-like gamma) constantly had increased expressions in the aortic tissues of AS mice, and CD79 always had decreased expression in the tissues (Figure 5). These DEGs may play important roles in AS pathogenesis.

Among the 5,414 cells in cluster 1, 5,209 cells were annotated as B cells (Figure 2 and Supplementary file 7), because Ms4a1 (CD20) and CD19, well-known markers of B cells, were among the top 10 markers of Cluster 1 (Figure 4). The mature naïve B-cell repertoire includes three well-defined populations of B1, B2 (follicular B, FOB), and marginal zone B (MZB) cells [10]. Marginal zone B and B1-cell-specific protein 1 (MZB1), Fc Mu receptor (Fcmr and TOSO), and BAFF receptor (BAFFR and TNFRSF13C) were also among the top 10 markers (Figure 4). MZB1 is highly expressed in MZB cells and B1 cells [11], BAFFR is the major receptor expressed in B1 cells [12], and defects in BAFFR expression prevent the formation of MZB cells [13]. Fcmr ablation reduces MZB cell numbers [14]. These studies suggest that cluster 1 was mainly composed of B1 cells and/or MZB cells with high expression of MZB1, Fcmr, and BAFFR. Among the 3,783 cells in cluster 2, 2,196 cells were annotated as T cells (Figure 2 and Supplementary file 7). CD3 and Il2rb (interleukin 2 receptor subunit beta, CD122, and IL15rb) are well-known markers of T cells and are among the top 10 markers of this cluster. Because CD8b1 as top 20 DEG also had increased expression as a marker in this cluster (Supplementary file 8 and GSE254708), cluster 2 was considered CD8+CD122+ T cells. In cluster 7, 2,230 of the 2,253 cells were identified as stromal cells (Figure 2 and Supplementary file 7). Among the top 20 DEGs, Myh11 (myosin heavy chain 11), acta2 (actin alpha 2 and smooth muscle), and Tagln (transgelin) were also marker genes of this cluster (Supplementary file 8 and GSE254708), and they have been reported as smooth muscle cell markers [15]. Thus, cluster 7 was more likely to be composed of smooth muscle cells with stromal features. In cluster 8, 967, 676, and 457 cells among 2,171 cells were annotated as stromal cells, endothelial cells, and fibroblasts, respectively (Figure 2 and Supplementary file 7). In cluster 14, 204 of the 406 cells in this cluster were identified as macrophages (Figure 2 and Supplementary file 7). Monocytes are divided into classical CD14++CD16−, intermediate CD14++CD16+, and nonclassical CD14+CD16++ monocytes [11]. CD16 (Fcgr3) was among the top 10 markers of this cluster and was only expressed in cluster 14. CD14, a well-known marker of monocytes, is a marker gene of cluster 14, but its expression level (avg log2FC = 2.82, gene diff = 10.96) was lower than that of CD16 (avg log2FC = 1.66, gene diff = 48.29) (Supplementary file 8 and GSE254708). Thus, the cells in cluster 14 were most likely nonclassical CD14+CD16++ monocytes. In cluster 16, 146 of the 295 cells showed a significant association with macrophages (Figure 2 and Supplementary file 7). Thus, these clusters were considered macrophages.

**Figure 6:**
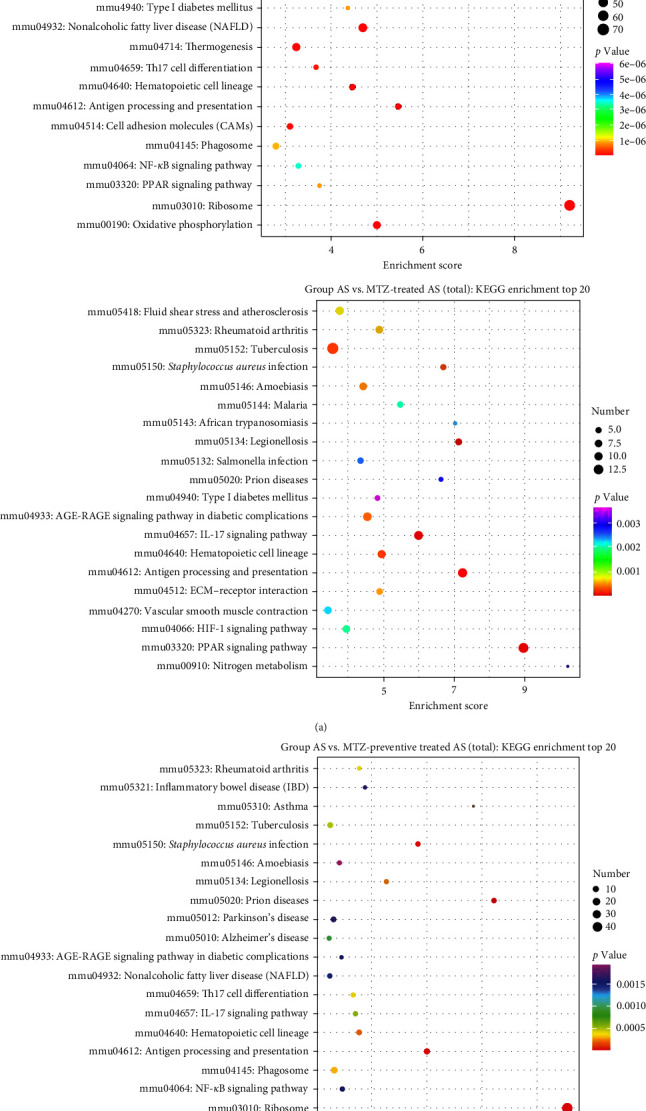
Bubble plot of pathway analysis in the mouse aorta based on the top 20 DEGs.

KEGG enrichment analysis was performed based on DEGs between the healthy group and the AS, AS with MTZ treatment, and AS with MTZ-preventive treatment groups. Pathways involved in antigen processing and presentation, hematopoietic cell lineage, rheumatoid arthritis, and *Staphylococcus aureus* infection were all enriched and activated in the above three comparative analyses, indicating that AS development and MTZ treatment are mediated by these three pathways (Figure 6).

The top 30 DEGs were analyzed by GO enrichment analysis, and GO entries with more than two different genes were screened out among the three classified genes (biological process, cellular component, and molecular function). According to the corresponding −log10*p* value of each entry, there were 10 items in descending order. The expression levels of genes related to the response to bacteria, the extracellular space, and the cell surface were consistently increased or decreased, as determined by comparing the expression profiles between AS mice and normal mice, AS mice and MTZ-treated mice, and AS mice and MTZ-preventive treated mice (Supplementary file 9).

### 3.3. The Percentage of CD8+CD183 (CXCR3)+ T Cells Was Decreased in AS Patients

**Figure 7:**
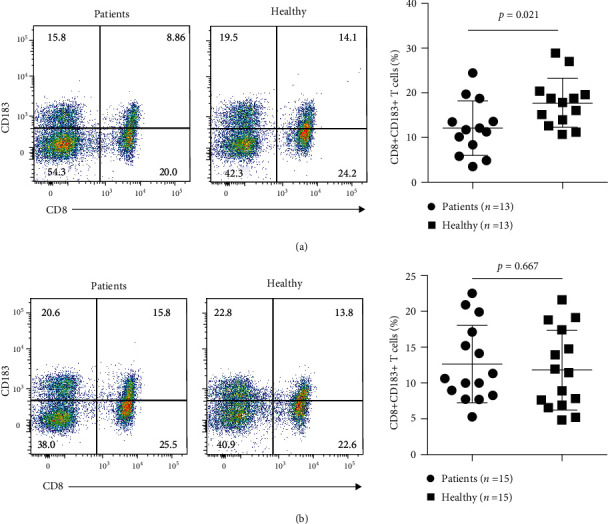
The CD8+CD183+ T-cell level in the peripheral blood of AS patients was determined using flow cytometry. (a) CD8+CD183+ T-cell levels in healthy controls and newly diagnosed AS patients without treatment. (b) CD8+CD183+ T-cell levels in healthy controls and AS patients receiving various treatments. Significantly decreased levels of CD8+CD183+ T cells were exclusively detected in the peripheral blood of patients newly diagnosed with AS.

We measured the levels of human CD8+CD183+ T cells, the counterpart of murine CD8+CD122+ cells, in peripheral blood samples from patients with AS using flow cytometry. Compared with those in healthy subjects, the numbers of CD8+CD183+ T cells were significantly lower in patients (*n* = 13) newly diagnosed with AS (*p* = 0.021). There were no significant changes in CD8 + CD183+ T-cell levels between AS patients (*n* = 16) receiving anti-AS treatments and healthy control subjects (*p* = 0.921) (Figure 7 and Supplementary file 10). This result indicated a significant decrease in CD8+CD183+ T cells in AS patients without any treatment.

## 4. Discussion

In the present study, AS model animals that received MTZ treatment or MTZ-preventive treatment exhibited significantly less plaque formation and thinning of the heart aorta intima than did AS model animals without MTZ treatment, while they exhibited significantly greater serum HDL and NO levels and lower LDL levels. The above findings showed that MTZ had a therapeutic effect on AS, as we previously observed [5]. Moreover, the levels of IL-6, IFN-*γ*, TNF-*α*, and IL-1*β* were decreased, and the Treg proportion was elevated in AS mice after MTZ treatment. Many studies demonstrated that decreased proportions of Treg cells and dysfunction of Treg cell contribute to AS pathogenesis. Treg cells can suppress excessive immune responses to exert atheroprotective effects [16, 17]. Our result suggests that MTZ exerts a therapeutic effect on AS by suppressing proinflammatory cytokine production and increasing the Treg proportion.

The aortic tissues of AS mice were analyzed using single-cell sequencing, and 20 cell clusters were detected in the tissues. The proportions of clusters 1, 2, and 7 in the aorta samples were associated with health, while the proportions of clusters 8, 14, and 16 were associated with AS. Cluster 1 included B1 and/or MZB cells. B cells contribute to AS through subset-specific mechanisms [18, 19]. Many studies have demonstrated that B1 cells are an atheroprotective subset that secretes protective IgM antibodies to scavenge deleterious molecules that promote AS [20], and MZB cells can proliferate and differentiate into IgM plasmablasts to protect against AS [21]. Nr4A1 (nuclear receptor subfamily four group A member 1, NUR77) and Ccr7 (C-C motif chemokine receptor 7) were the top 20 DEGs between normal and AS mice and between AS and MTZ-preventive AS mice, and they were also marker genes of Cluster 1 (Supplementary file 8 and GSE254708). The expression of these genes increased in the healthy control mice and the MTZ-preventive treated mice and decreased in the AS model mice in this cluster (Figure 5). Deletion of Nr4A1 in MZB cells stimulates AS development [22], and Nr4A1 (−/−) mice develop substantial AS pathologies [23]; Ccr7−/− mice exhibit a partial reduction in the number of peritoneal B1 cells [24]. The above studies support that cluster 1 was composed mainly of B1 cells and/or MZB cells with high expression of Nr4A1 and Ccr7 to impede AS progression. This cluster had a high proportion in the aorta of normal mice and AS mice following MTZ treatment to maintain atheroprotective IgM production.

Cluster 2 was annotated as CD8+CD122+ T cells. This T-cell subtype is a Treg-like cell subtype and is more potent in immunosuppression than conventional Treg cells [25]. Nr4A1 was also a gene marker of cluster 2 (Supplementary file 8 and GSE254708) and had increased expression levels in healthy control mice and in MTZ-preventive treated mice (Figure 5). Deletion of Nr4A1 overcame T-cell tolerance and abrogated Treg cell suppressive activities [26]. A reduction in or dysfunction of Treg cells mainly contributes to AS by impairing immune suppression and promoting autoimmunity [16]. The above findings support that cluster 2 was composed mainly of atheroprotective CD8+CD122+ T cells with high expression of Nr4A1 that exerted immunosuppressive effects and atheroprotective effects. This cluster had a high proportion in the aorta of normal mice and AS mice following MTZ treatment to suppress AS immunity.

Cluster 7 was considered smooth muscle cells. No studies have reported the associations of the top 10 markers with AS or biocalcification. Among the top 20 DEGs, Tpm2 was a gene marker of cluster 7 (Supplementary file 8 and GSE254708). It had low expression in the AS model mice and at high levels in AS model mice with MTZ treatment (Figure 5). The expression level of Tpm2 is lower in human AS artery tissue and AS model mice [27], and Tpm2 was considered a potential target for the clinical treatment of AS [28]. Thus, cluster 7, with high Tpm2 expression, was composed mainly of smooth muscle cells with stromal features. This cluster had a high proportion in the aortas of AS mice following MTZ treatment and can play an atheroprotective role.

Cluster 8 included stromal cells, endothelial cells, and fibroblasts. Retn (Resistin) was the top 10 markers of cluster 8 and was among the top 20 DEGs according to comparisons of expression profiles between normal control mice and AS mice and between AS mice and MTZ-treated mice. Retn is expressed in endothelial cells, vessel smooth muscle cells, and macrophages in AS patients [29], and its expression is strongly associated with inflammation and calcification in coronary artery diseases [30, 31]. In addition, Retn mediates cardiac fibroblast-to-myofibroblast differentiation, potentially leading to the stimulation of cardiac fibrosis [32]. Apoc1 is also one of the top 10 markers of this cluster, and it can aggravate AS development in ApoE-knockout mice [33]. Among the top 20 DEGs, Lcn2, Mt1, Serpina3n (serpin family A member 3), Lgals3 (galectin-3 or Gal-3), and LPL (lipoprotein lipase) were also marker genes of this cluster (Supplementary file 8 and GSE254708). These genes exhibited high expression in AS model mice and low expression in normal control, MTZ-treated, and MTZ-preventive treated mice (Figure 5). Lcn2 accelerates AS development [34]; MT polymorphisms have been identified and associated with AS [35]. Mt1 and Mt2 have been implicated in the process of angiogenesis in aortic endothelial cells [36, 37]; SerpinA3 is expressed in endothelial cells and medial smooth muscle cells in atherosclerotic lesions, and its expression is increased in lesions of Apoe−/− mice [38]; LPL mass and activity are significantly correlated with the extent of calcific atherosclerosis [39]; and Lgals3 is considered a biomarker for the diagnosis of AS [40]. Lgals3 levels were also significantly increased in patients with calcific aortic valve disease and stimulated calcification in patients with AS [41]. An inhibitor of Lgals3 reduced AS plaque volume [42]. These studies suggest that cluster 8 cells are endothelial cells or fibroblasts with stromal features. This cluster had a high proportion in the AS aorta and may exert proatherogenic, proinflammatory, and calcification-inducing effects with high expression of Retn, Apoc1, Lcn2, Mt1, Serpina3, Lpl, and Lgals3.

Cluster 14 is considered nonclassical CD14+CD16++ monocytes. CD16 was among the top 10 markers of this cluster. CD16 has been reported to be associated with AS [43]. The transmembrane immune signaling adaptor (Tyrobp), Mt1, Lgals3, and CXCl2 genes were among the top 20 DEGs and were also marker genes of this cluster (Supplementary file 8 and GSE254708). These genes exhibited increased expression in AS model mice and decreased expression in normal control mice or AS model mice preventively treated with MTZ (Figure 5). Tyrobp is involved in the inflammatory response of macrophages. Tyrobp-deficient mice had longer survival times, ameliorated cardiac remodeling, and improved cardiac function [44]. Elevated expression of CXCL2 was detected in the plaques and peripheral blood of AS patients [45, 46]. Thus, cluster 14 was mainly composed of nonclassical CD14+CD16++ monocytes. This cluster had a high proportion in the aortas of AS mice and may stimulate inflammation and calcification in atherosclerotic processes by increasing the expression of Mt1, Tyrobp, Lgals3, and Cxcl2.

Cluster 16 was mainly composed of macrophages. Spp1, Mmp12 (matrix metallopeptidase 12), and C1qb (complement C1q b chain) were among the top 10 markers and the top 20 DEGs in this cluster. Spp1 is highly expressed in AS plaques, especially in macrophages and foam cells [47]. Spp1+ macrophages aggravate fibrosis in AS via OPN-CD44/integrin interactions [48]; MMP-12 is overexpressed in AS vascular tissues and calcified aortic valves and induces calcium deposit formation [49, 50]; and triggering receptor expressed on myeloid cells 2 (Trem2) is among the top 10 markers of cluster 16. Atherosclerotic plaques express Trem2 [51], and Trem2 mediates vascular calcification [52]. The top 20 DEGs, including Mt1, Cxcl2, Lpl, and Tyrobp, were also gene markers of this cluster (Supplementary file 8 and GSE254708). These genes exhibited increased expression in AS model mice and decreased expression in healthy control mice and in mice subjected to MTZ or MTZ-preventive treatment (Figure 5). Lgals3 is a potential mediator of AS [53]. Lgals3 is secreted by activated macrophages and is involved in cardiac fibrosis, cardiac tissue remodeling, and inflammation [54]. Upregulation of Lgals3 in macrophages induced diabetic vascular intimal calcification [55]. These studies suggested that cluster 16 consisted of proatherogenic Spp1+ macrophages and may stimulate calcification and inflammation by increasing the expression of Spp1, Mmp-12, Trem2, Mt1, Lgals3, Cxcl2, and Lpl. This cluster had a high proportion in the aortic tissues of AS mice.

Human CD8+CD183 (CXCR3)+ T cells are the counterparts of murine CD8+CD122 cells and have the same immunosuppressive function as Tregs [56]. In the present study, the number of CD8+CD183+ T cells was significantly decreased in the peripheral blood of patients newly diagnosed with AS. There were no significant changes in CD8+CD183+ T-cell proportions between AS patients receiving various treatments and healthy control subjects. This is the first report on the involvement of CD8+CD183+ T cells in AS. This finding suggested that a decreased number of CD8+CD183+ T cells may disrupt immunosuppression in AS. Although other types of T cells are present in AS and play proatherogenic or atheroprotective roles [57], our study did not find alternative expression of other T-cell types in AS mice or AS patients.

AS is considered an autoimmune disease [58, 59]. Plaque calcification develops in an inflammation-dependent manner in AS [60]. In the present study, Lgals3, Retn, and Mmp-12, which were among the top DEGs and were mainly expressed in clusters 8, 14 and 16, exhibited increased expression in AS mice and decreased expression in healthy control mice and AS model mice following MTZ treatment. These genes have both calcification-stimulating and proinflammatory functions, as described above and based on previous studies, indicating that calcification and inflammation are integrated during the AS process. Thus, inhibiting calcification with MTZ in our study can simultaneously suppress chronic inflammation in AS patients, which can explain why MTZ, an anticalcification drug, can suppress inflammation and AS progression. Our results suggest that the increased expression of Lgals3, Retn, and Mmp-12 in clusters 8, 14, and 16 promoted AS inflammation and calcification, while decreased proportions of B1/MZB cells and CD8+CD122 cells or CD8+CD183 (CXCR3)+ T cells aggravated AS by impairing immune suppression and atheroprotective mechanisms.

The present study detected pathways associated with antigen processing and presentation, hematopoietic cell lineage, rheumatoid arthritis, and *S. aureus* infection in aortic samples based on the top DEGs. AS is a chronic disease, and the interaction between innate and adaptive immune cells enhances the AS-associated immune-inflammatory response. Antigen processing and presentation are key to the immune response. Immune cells that self-epitope often interrupt immune tolerance, which contributes to atherogenesis [61, 62]. Multiple leukocyte lineages are generated within the bone marrow. Growing evidence indicates that bone marrow–derived cells are deeply involved in atherogenesis [63]. Bone marrow–derived vascular progenitor cells play an important role in AS pathogenesis [64]. Additionally, the number of AS patients with RA is much greater than that in the general population, and atherosclerotic lesions develop much more rapidly in AS patients with RA [65]. Proinflammatory cytokines, RA-related autoantibodies, and endothelial dysfunction accelerate the pathology of both RA and AS [66]. The above studies supported our findings.

## 5. Conclusions

This study revealed that MTZ can treat AS in an animal model by increasing the proportions of Treg cells, B-1/MZB cells with high expression of Nr4A1 and Ccr7, CD8+CD122+ Treg-like cells with high Nr4A1 expression, and smooth muscle cells with high expression of Tpm2 to suppress the immune response and exert atheroprotective effect. Moreover, MTZ decreased the proportions of endothelial cells or fibroblast types with high expression of Retn, Apoc1, Lcn2, Mt1, Serpina3, Lpl, and Lgals3; nonclassical CD14+CD16++ monocytes with high expression of Mt1, Tyrobp, Lgals3, and Cxcl2; and Spp1+ macrophages with high expression of Mmp-12, Trem2, Mt1, Lgals3, Cxcl2, and Lpl to exert anti-AS effects by suppressing inflammation, calcification, and tissue remodeling. MTZ can be used for AS therapy by increasing immune suppression and inhibiting calcification and inflammation-regulating gene expressions.

## Data Availability

The data used to support the findings of this study are included within the article. The raw single-cell transcriptome sequencing data have been deposited in the NCBI GEO repository (accession number: GSE254708).

## Supplementary Materials

Supplementary 1 File 1: Clinical information of AS patients.

Supplementary 2 File 2: Biochemical examination in mouse peripheral blood. The biochemistry of AS model mice was clearly changed, and some indexes were remarkably restored after MTZ treatment. AI, apolipoprotein; HDL, high-density lipoprotein; LDL, low-density lipoprotein; TC, total cholesterol; TG, triglyceride; and NO, nitric oxide.  ^*∗*^ indicates *p* < 0.05,  ^*∗∗*^ indicates *p* < 0.01,  ^*∗∗∗*^ indicates *p* < 0.001, and  ^*∗∗∗∗*^ indicates *p* < 0.0001.

Supplementary 3 File 3: Routine mouse blood test in mouse peripheral blood. The hemogram of AS model mice was clearly changed, and some indexes were remarkably affected after MTZ treatment. EOS, eosinophils; Lym, lymphocytes; Mon, monocytes; Neu, neutrophils; and WBC, white blood cells.  ^*∗*^ indicates *p* < 0.05,  ^*∗∗*^ indicates *p* < 0.01,  ^*∗∗∗*^ indicates *p* < 0.001, and  ^*∗∗∗∗*^ indicates *p* < 0.0001.

Supplementary 4 File 4: Measurement of serum cytokine levels in mouse peripheral blood using flow cytometry. Cytokine production in AS model mice was clearly changed, and their levels went to normal after MTZ treatment, especially IL-6, IFN-*γ*, TNF-*α*, and IL-1*β* levels.  ^*∗*^ indicates *p* < 0.05,  ^*∗∗*^ indicates *p* < 0.01,  ^*∗∗∗*^ indicates *p* < 0.001, and  ^*∗∗∗∗*^ indicates *p* < 0.0001.

Supplementary 5 File 5: Evaluation of lymphocyte subtype proportions in total lymphocytes in mouse peripheral blood using flow cytometry. The proportion of Treg cells in AS model mice was clearly decreased, and the proportion of Treg cells was markedly elevated after MTZ treatment.  ^*∗*^ indicates *p* < 0.05,  ^*∗∗*^ indicates *p* < 0.01, and  ^*∗∗∗*^ indicates *p* < 0.001.

Supplementary 6 File 6: Reduced dimensional clustering analysis of gene expressions in mouse aorta tissues. AS model mice with or without MTZ treatment were analyzed via single-cell sequencing. Twenty cell clusters were identified, and each cluster is distinguished by a different color. The abscissa and ordinate represent the first and second principal components of the reduced dimension, respectively. Each point in the diagram represents a cell. UMAP, uniform manifold approximation and projection.

Supplementary 7 File 7: Cell typing statistics based on the association of expression profiles in target cells with Mouseref immgen database.

Supplementary 8 File 8: top 20 DEGs and cluster marker genes. Those genes that were among the top 20 DEGs and cluster marker genes are shown in the table.

Supplementary 9 File 9: GO analysis of the top 30 DEGs. The expression levels of genes that were enriched in response bacteria, extracellular space, and the cell surface were altered in the aortic tissues of AS mice.

Supplementary 10 File 10: Gating strategy for CD8+CXCR3+ T cells. Freshly isolated peripheral monocytes were gated on single CD45+ lymphocytes. CD3+ T cells were further gated, and the CD8+CXCR3+ T-cell population was analyzed.
