# Supplementary material for: Methazolamide Can Treat Atherosclerosis by Increasing Immunosuppressive Cells and Decreasing Expressions of Genes Related to Proinflammation, Calcification, and Tissue Remodeling
Source: J Immunol Res. 2024 Jul 23;2024:5009637. doi: 10.1155/2024/5009637 (PMC11288698; doi:10.1155/2024/5009637)
Supplement: Supplementary 1 — File 1: Clinical information of AS patients. [file 5009637.f1.docx]

**Clinical information of AS patients**

| **Number** | **Gender** | **Age** | **Treatment** |
| --- | --- | --- | --- |
| 1 | Female | 84 | Treated |
| 2 | Female | 65 | Newly Diagnosed |
| 3 | Male | 81 | Treated |
| 4 | Female | 72 | Newly Diagnosed |
| 5 | Male | 68 | Newly Diagnosed |
| 6 | Female | 50 | Treated |
| 7 | Male | 55 | Treated |
| 8 | Female | 88 | Newly Diagnosed |
| 9 | Male | 77 | Treated |
| 10 | Male | 49 | Treated |
| 11 | Male | 60 | Treated |
| 12 | Male | 63 | Newly Diagnosed |
| 13 | Female | 59 | Treated |
| 14 | Male | 86 | Treated |
| 15 | Female | 62 | Treated |
| 16 | Female | 72 | Newly Diagnosed |
| 17 | Female | 49 | Newly Diagnosed |
| 18 | Male | 61 | Treated |
| 19 | Male | 63 | Treated |
| 20 | Male | 66 | Newly Diagnosed |
| 21 | Male | 87 | Treated |
| 22 | Male | 43 | Treated |
| 23 | Female | 63 | Newly Diagnosed |
| 24 | Male | 73 | Newly Diagnosed |
| 25 | Female | 72 | Newly Diagnosed |
| 26 | Female | 85 | Newly Diagnosed |
| 27 | Female | 72 | Newly Diagnosed |
| 28 | Female | 64 | Treated |
