# Supplementary figures and images for: Methazolamide Can Treat Atherosclerosis by Increasing Immunosuppressive Cells and Decreasing Expressions of Genes Related to Proinflammation, Calcification, and Tissue Remodeling
Source: J Immunol Res. 2024 Jul 23;2024:5009637. doi: 10.1155/2024/5009637 (PMC11288698; doi:10.1155/2024/5009637)

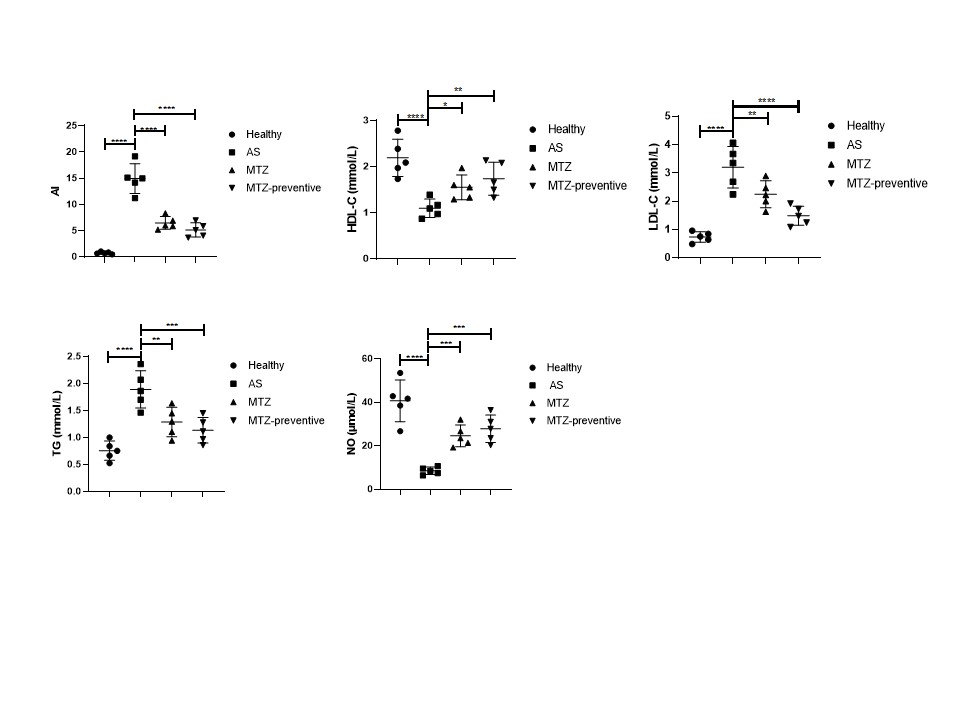

Supplement: Supplementary 2 — File 2: Biochemical examination in mouse peripheral blood. The biochemistry of AS model mice was clearly changed, and some indexes were remarkably restored after MTZ treatment. AI, apolipoprotein; HDL, high-density lipoprotein; LDL, low-density lipoprotein; TC, total cholesterol; TG, triglyceride; and NO, nitric oxide. ∗ indicates p < 0.05, ∗∗ indicates p < 0.01, ∗∗∗ indicates p < 0.001, and ∗∗∗∗ indicates p < 0.0001. [file 5009637.f2.jpg]

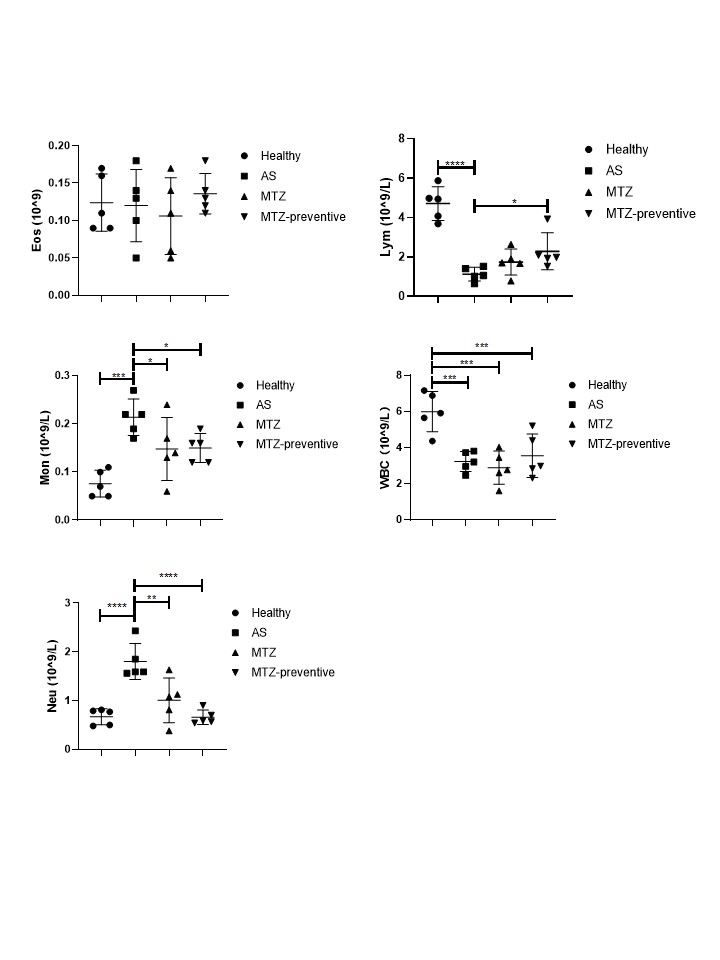

Supplement: Supplementary 3 — File 3: Routine mouse blood test in mouse peripheral blood. The hemogram of AS model mice was clearly changed, and some indexes were remarkably affected after MTZ treatment. EOS, eosinophils; Lym, lymphocytes; Mon, monocytes; Neu, neutrophils; and WBC, white blood cells. ∗ indicates p < 0.05, ∗∗ indicates p < 0.01, ∗∗∗ indicates p < 0.001, and ∗∗∗∗ indicates p < 0.0001. [file 5009637.f3.jpg]

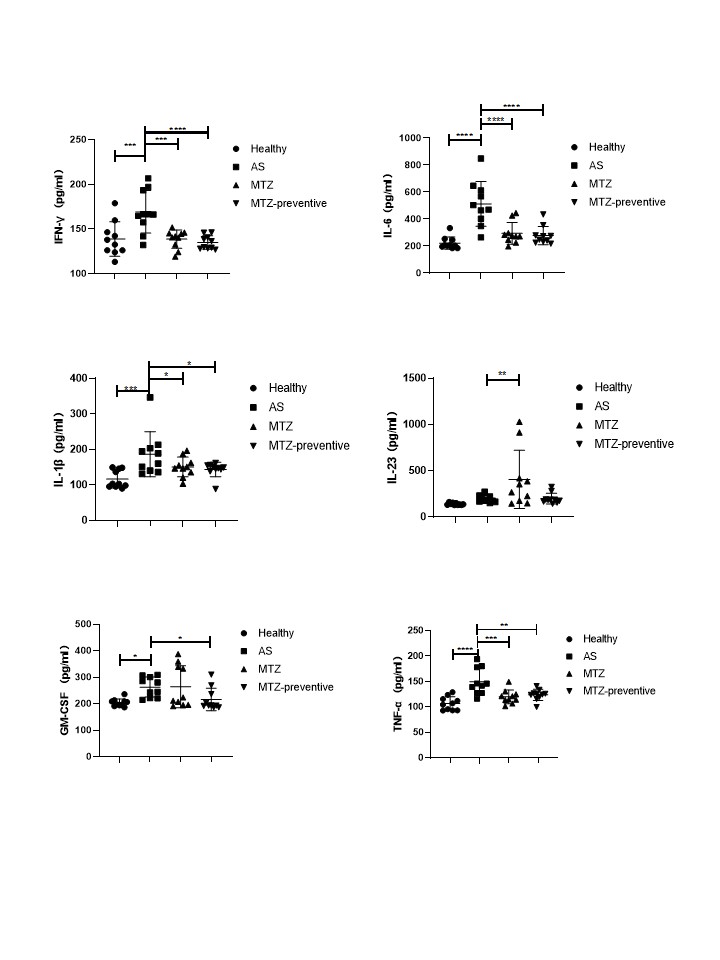

Supplement: Supplementary 4 — File 4: Measurement of serum cytokine levels in mouse peripheral blood using flow cytometry. Cytokine production in AS model mice was clearly changed, and their levels went to normal after MTZ treatment, especially IL-6, IFN-γ, TNF-α, and IL-1β levels. ∗ indicates p < 0.05, ∗∗ indicates p < 0.01, ∗∗∗ indicates p < 0.001, and ∗∗∗∗ indicates p < 0.0001. [file 5009637.f4.jpg]

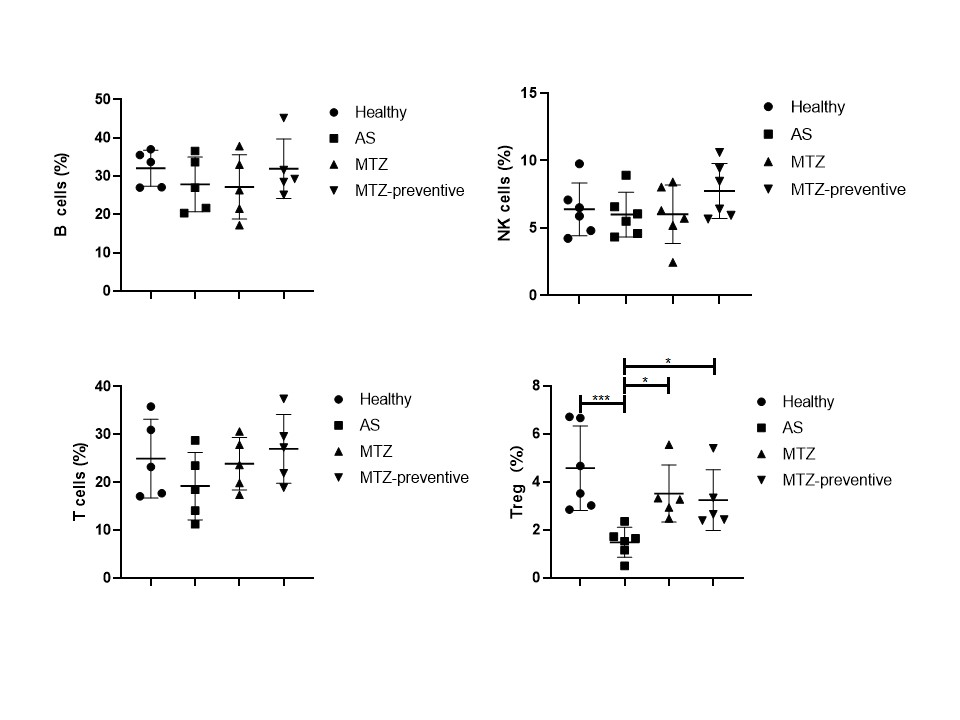

Supplement: Supplementary 5 — File 5: Evaluation of lymphocyte subtype proportions in total lymphocytes in mouse peripheral blood using flow cytometry. The proportion of Treg cells in AS model mice was clearly decreased, and the proportion of Treg cells was markedly elevated after MTZ treatment. ∗ indicates p < 0.05, ∗∗ indicates p < 0.01, and ∗∗∗ indicates p < 0.001. [file 5009637.f5.jpg]

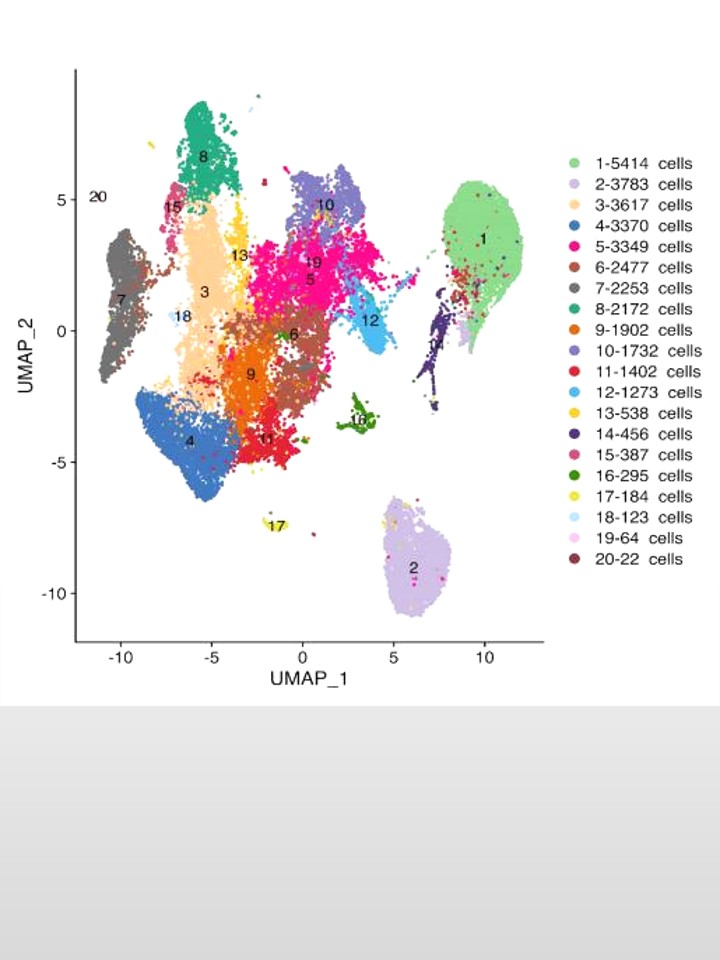

Supplement: Supplementary 6 — File 6: Reduced dimensional clustering analysis of gene expressions in mouse aorta tissues. AS model mice with or without MTZ treatment were analyzed via single-cell sequencing. Twenty cell clusters were identified, and each cluster is distinguished by a different color. The abscissa and ordinate represent the first and second principal components of the reduced dimension, respectively. Each point in the diagram represents a cell. UMAP, uniform manifold approximation and projection. [file 5009637.f6.jpg]

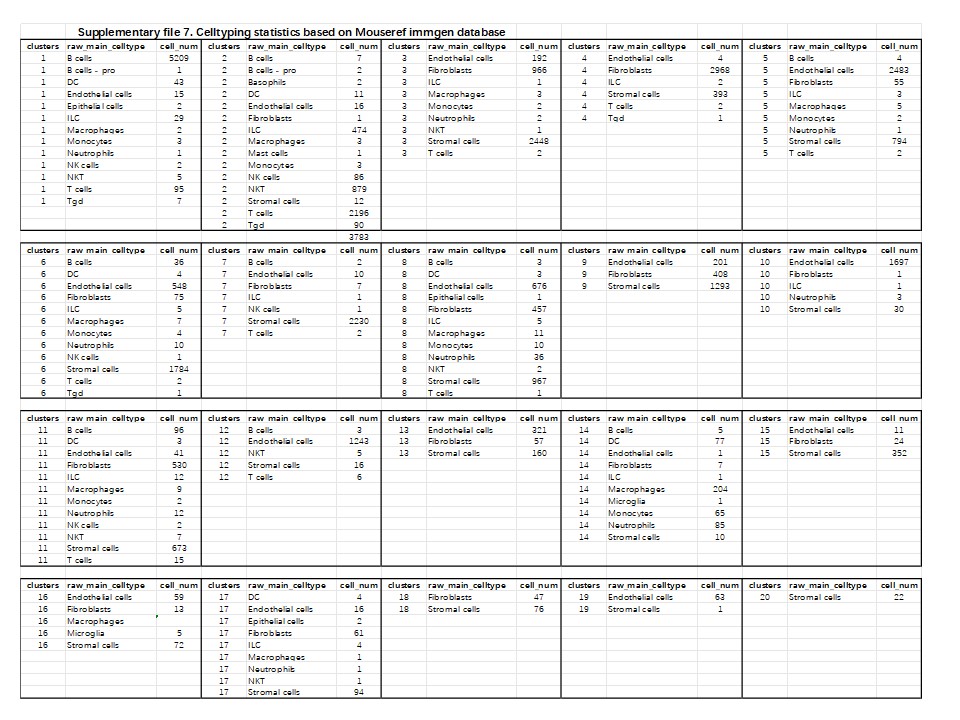

Supplement: Supplementary 7 — File 7: Cell typing statistics based on the association of expression profiles in target cells with Mouseref immgen database. [file 5009637.f7.jpg]

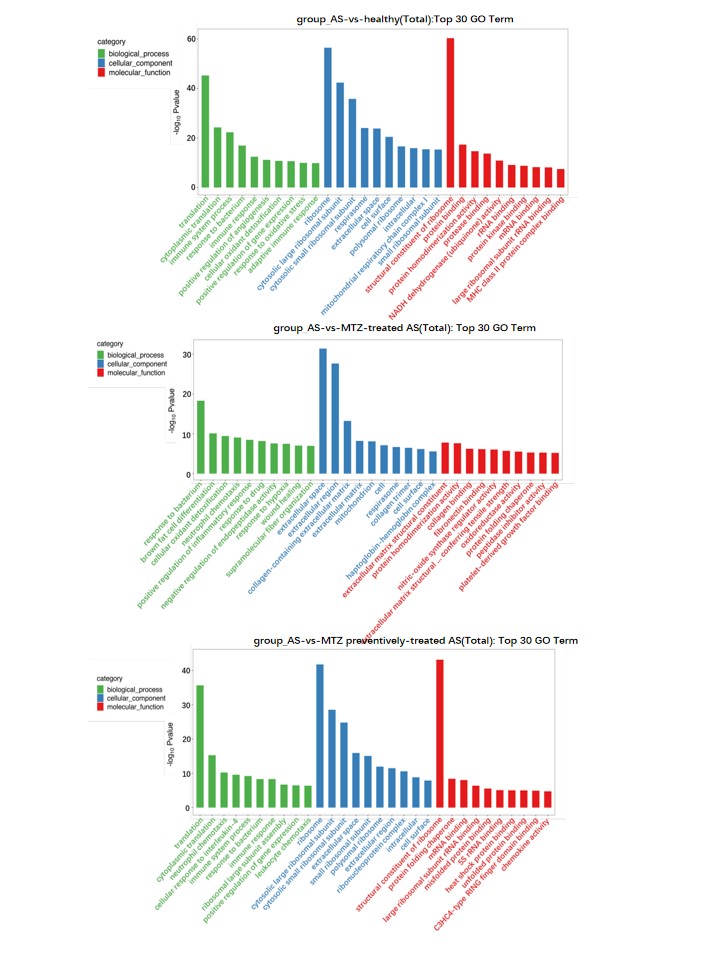

Supplement: Supplementary 9 — File 9: GO analysis of the top 30 DEGs. The expression levels of genes that were enriched in response bacteria, extracellular space, and the cell surface were altered in the aortic tissues of AS mice. [file 5009637.f9.jpg]

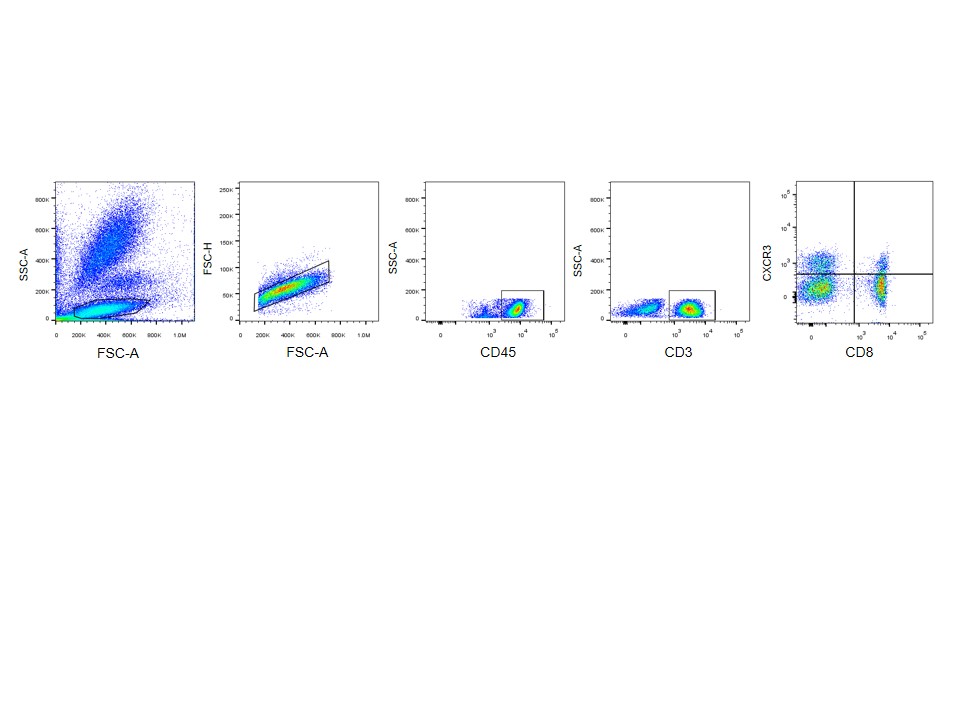

Supplement: Supplementary 10 — File 10: Gating strategy for CD8+CXCR3+ T cells. Freshly isolated peripheral monocytes were gated on single CD45+ lymphocytes. CD3+ T cells were further gated, and the CD8+CXCR3+ T-cell population was analyzed. [file 5009637.f10.jpg]
