# Supplementary material for: Methazolamide Can Treat Atherosclerosis by Increasing Immunosuppressive Cells and Decreasing Expressions of Genes Related to Proinflammation, Calcification, and Tissue Remodeling
Source: J Immunol Res. 2024 Jul 23;2024:5009637. doi: 10.1155/2024/5009637 (PMC11288698; doi:10.1155/2024/5009637)
Supplement: Supplementary 8 — File 8: top 20 DEGs and cluster marker genes. Those genes that were among the top 20 DEGs and cluster marker genes are shown in the table. [file 5009637.f8.docx]

**Supplementary file 8. Top 20 DEGs and cluster marker genes**

| **gene** | **p_val** | **avg_log2FC** | **pct.1** | **pct.2** | **p_val_adj** | **cluster** | **gene_diff** |
| --- | --- | --- | --- | --- | --- | --- | --- |
| Cd74 | 0 | 3.976324394 | 0.999 | 0.406 | 0 | 1 | 2.461 |
| H2-Eb1 | 0 | 3.522713415 | 0.986 | 0.208 | 0 | 1 | 4.74 |
| H2-Aa | 0 | 3.555935157 | 0.984 | 0.208 | 0 | 1 | 4.731 |
| H2-Ab1 | 0 | 3.39820326 | 0.98 | 0.19 | 0 | 1 | 5.158 |
| Cd79a | 0 | 3.716601064 | 0.946 | 0.099 | 0 | 1 | 9.556 |
| Cd37 | 0 | 2.524805608 | 0.791 | 0.124 | 0 | 1 | 6.379 |
| Hsp90aa1 | 0 | 1.76126658 | 0.958 | 0.72 | 0 | 1 | 1.331 |
| Dusp2 | 0 | 2.104579753 | 0.821 | 0.253 | 0 | 1 | 3.245 |
| Cd52 | 0 | 1.841929091 | 0.802 | 0.23 | 0 | 1 | 3.487 |
| Rel | 0 | 2.353499256 | 0.685 | 0.149 | 0 | 1 | 4.597 |
| Nr4a1 | 0 | 1.605929309 | 0.868 | 0.586 | 0 | 1 | 1.481 |
| Nfkbid | 0 | 2.091821032 | 0.655 | 0.13 | 0 | 1 | 5.038 |
| Hspa8 | 0 | 0.950514543 | 0.983 | 0.923 | 0 | 1 | 1.065 |
| Ighm | 0 | 1.724873598 | 0.579 | 0.065 | 0 | 1 | 8.908 |
| Ccr7 | 0 | 2.33893803 | 0.565 | 0.071 | 0 | 1 | 7.958 |
| Junb | 0 | 0.963464653 | 0.98 | 0.911 | 0 | 1 | 1.076 |
| Eef1a1 | 0 | 0.649522556 | 0.996 | 0.969 | 0 | 1 | 1.028 |
| Tmsb10 | 0 | 0.623223297 | 0.998 | 0.921 | 0 | 1 | 1.084 |
| Rilpl2 | 0 | 2.038304809 | 0.612 | 0.181 | 0 | 1 | 3.381 |
| Hspa1b | 2.61E-178 | 1.401681891 | 0.473 | 0.32 | 8.42E-174 | 1 | 1.478 |
| Hspa1a | 1.80E-123 | 1.299367371 | 0.486 | 0.367 | 5.80E-119 | 1 | 1.324 |
| Tmsb10 | 0 | 1.834077045 | 1 | 0.924 | 0 | 2 | 1.082 |
| Eef1a1 | 0 | 0.821180331 | 0.99 | 0.971 | 0 | 2 | 1.02 |
| Cd52 | 0 | 1.453198608 | 0.812 | 0.259 | 0 | 2 | 3.135 |
| Junb | 0 | 0.955810524 | 0.975 | 0.915 | 0 | 2 | 1.066 |
| Hspa8 | 0 | 0.838956225 | 0.979 | 0.927 | 0 | 2 | 1.056 |
| Dusp2 | 0 | 1.24031101 | 0.732 | 0.293 | 0 | 2 | 2.498 |
| Hsp90aa1 | 0 | 0.679942435 | 0.918 | 0.738 | 0 | 2 | 1.244 |
| Selplg | 0 | 1.359480891 | 0.411 | 0.048 | 0 | 2 | 8.562 |
| Nfkbid | 0 | 0.895255898 | 0.505 | 0.176 | 0 | 2 | 2.869 |
| Cd8b1 | 0 | 1.448192071 | 0.284 | 0.016 | 0 | 2 | 17.75 |
| Cd37 | 1.63E-100 | 0.038126523 | 0.408 | 0.206 | 5.25E-96 | 2 | 1.981 |
| Hspa1b | 7.23E-99 | 0.529217218 | 0.498 | 0.325 | 2.33E-94 | 2 | 1.532 |
| Nr4a1 | 3.69E-71 | 0.327323578 | 0.766 | 0.613 | 1.19E-66 | 2 | 1.25 |
| Rilpl2 | 2.11E-32 | 0.055365731 | 0.345 | 0.236 | 6.80E-28 | 2 | 1.462 |
| Tagln | 0 | 3.803803674 | 0.999 | 0.498 | 0 | 7 | 2.006 |
| Acta2 | 0 | 3.799931756 | 1 | 0.452 | 0 | 7 | 2.212 |
| Myl9 | 0 | 3.693990972 | 0.995 | 0.319 | 0 | 7 | 3.119 |
| Tpm2 | 0 | 3.568012361 | 0.994 | 0.306 | 0 | 7 | 3.248 |
| Myl6 | 0 | 2.776113493 | 0.998 | 0.769 | 0 | 7 | 1.298 |
| Myh11 | 0 | 3.00066044 | 0.962 | 0.234 | 0 | 7 | 4.111 |
| Nenf | 0 | 0.918179866 | 0.848 | 0.369 | 0 | 7 | 2.298 |
| Rcn3 | 1.16E-219 | 0.555543611 | 0.569 | 0.227 | 3.74E-215 | 7 | 2.507 |
| Phpt1 | 1.74E-187 | 0.490825609 | 0.376 | 0.133 | 5.63E-183 | 7 | 2.827 |
| Hp1bp3 | 6.84E-86 | 0.274972376 | 0.341 | 0.158 | 2.21E-81 | 7 | 2.158 |
| Chpt1 | 5.15E-84 | 0.054987351 | 0.268 | 0.111 | 1.66E-79 | 7 | 2.414 |
| Pnpla2 | 3.02E-52 | 0.253257331 | 0.436 | 0.252 | 9.74E-48 | 7 | 1.73 |
| Hspa1a | 7.42E-33 | 0.008951276 | 0.56 | 0.373 | 2.40E-28 | 7 | 1.501 |
| C1qbp | 1.53E-29 | 0.049861909 | 0.3 | 0.18 | 4.93E-25 | 7 | 1.667 |
| Hp | 0 | 4.062815438 | 0.985 | 0.201 | 0 | 8 | 4.9 |
| Mt1 | 0 | 2.446613184 | 0.999 | 0.615 | 0 | 8 | 1.624 |
| Cyp2e1 | 0 | 3.52302721 | 0.863 | 0.089 | 0 | 8 | 9.697 |
| Pnpla2 | 0 | 2.679414063 | 0.882 | 0.223 | 0 | 8 | 3.955 |
| Cidec | 0 | 3.002988534 | 0.79 | 0.056 | 0 | 8 | 14.107 |
| Lrg1 | 0 | 2.47366188 | 0.86 | 0.216 | 0 | 8 | 3.981 |
| Mt2 | 0 | 1.747053837 | 0.882 | 0.332 | 0 | 8 | 2.657 |
| Retn | 0 | 2.959046114 | 0.659 | 0.041 | 0 | 8 | 16.073 |
| Car3 | 0 | 3.54804403 | 0.65 | 0.071 | 0 | 8 | 9.155 |
| Serpina3n | 0 | 1.642974862 | 0.723 | 0.177 | 0 | 8 | 4.085 |
| Lpl | 0 | 2.015331492 | 0.662 | 0.126 | 0 | 8 | 5.254 |
| Chpt1 | 0 | 2.076835943 | 0.581 | 0.09 | 0 | 8 | 6.456 |
| Lcn2 | 0 | 1.324100406 | 0.488 | 0.099 | 0 | 8 | 4.929 |
| Cd14 | 0 | 0.925905812 | 0.263 | 0.037 | 0 | 8 | 7.108 |
| AY036118 | 4.77E-204 | 0.747153467 | 0.998 | 0.962 | 1.54E-199 | 8 | 1.037 |
| Chp1 | 3.59E-155 | 0.71324627 | 0.321 | 0.12 | 1.16E-150 | 8 | 2.675 |
| C1qbp | 4.36E-57 | 0.426089802 | 0.32 | 0.178 | 1.41E-52 | 8 | 1.798 |
| Nhp2 | 8.25E-41 | 0.280830012 | 0.273 | 0.156 | 2.66E-36 | 8 | 1.75 |
| Lgals3 | 2.66E-28 | 0.017238727 | 0.27 | 0.166 | 8.59E-24 | 8 | 1.627 |
| Tyrobp | 0 | 4.314156153 | 0.934 | 0.062 | 0 | 14 | 15.065 |
| Fcer1g | 0 | 4.180864283 | 0.921 | 0.054 | 0 | 14 | 17.056 |
| Alox5ap | 0 | 3.925872055 | 0.818 | 0.033 | 0 | 14 | 24.788 |
| Lyz2 | 0 | 4.978486515 | 0.748 | 0.05 | 0 | 14 | 14.96 |
| Wfdc17 | 0 | 4.234591293 | 0.645 | 0.029 | 0 | 14 | 22.241 |
| Cd14 | 0 | 2.820519346 | 0.493 | 0.045 | 0 | 14 | 10.956 |
| C1qb | 0 | 4.312291239 | 0.399 | 0.025 | 0 | 14 | 15.96 |
| Fcgr3 （CD16） | 0 | 1.656947323 | 0.338 | 0.007 | 0 | 14 | 48.286 |
| Lgals3 | 3.39E-273 | 3.10658618 | 0.726 | 0.165 | 1.09E-268 | 14 | 4.4 |
| Cxcl2 | 2.53E-205 | 5.143070785 | 0.493 | 0.094 | 8.17E-201 | 14 | 5.245 |
| Selplg | 6.62E-148 | 1.505151534 | 0.428 | 0.083 | 2.14E-143 | 14 | 5.157 |
| Cd52 | 4.64E-141 | 1.682262812 | 0.807 | 0.312 | 1.50E-136 | 14 | 2.587 |
| H2-Ab1 | 1.66E-77 | 2.429539188 | 0.621 | 0.309 | 5.35E-73 | 14 | 2.01 |
| H2-Aa | 1.42E-63 | 2.062445115 | 0.601 | 0.325 | 4.59E-59 | 14 | 1.849 |
| H2-Eb1 | 2.73E-58 | 2.144800327 | 0.586 | 0.326 | 8.80E-54 | 14 | 1.798 |
| Cd74 | 3.26E-49 | 1.818507127 | 0.704 | 0.495 | 1.05E-44 | 14 | 1.422 |
| Rilpl2 | 1.18E-16 | 0.58775734 | 0.423 | 0.246 | 3.80E-12 | 14 | 1.72 |
| Tagln2 | 5.73E-16 | 0.683698166 | 0.566 | 0.404 | 1.85E-11 | 14 | 1.401 |
| Nhp2 | 3.23E-07 | 0.167887526 | 0.261 | 0.162 | 0.010420016 | 14 | 1.611 |
| C1qb | 0 | 4.004927394 | 0.871 | 0.023 | 0 | 16 | 37.87 |
| Lyz2 | 0 | 4.466928149 | 0.824 | 0.053 | 0 | 16 | 15.547 |
| Fcer1g | 0 | 2.942468 | 0.793 | 0.059 | 0 | 16 | 13.441 |
| Spp1 | 0 | 3.839219029 | 0.763 | 0.03 | 0 | 16 | 25.433 |
| Tyrobp | 0 | 2.665381118 | 0.769 | 0.067 | 0 | 16 | 11.478 |
| Alox5ap | 0 | 2.268428054 | 0.546 | 0.039 | 0 | 16 | 14 |
| Trem2 | 0 | 2.502131155 | 0.444 | 0.008 | 0 | 16 | 55.5 |
| Wfdc17 | 0 | 2.403479755 | 0.447 | 0.033 | 0 | 16 | 13.545 |
| Mmp12 | 0 | 3.545557945 | 0.322 | 0.002 | 0 | 16 | 161 |
| Lgals3 | 9.54E-288 | 3.487716471 | 0.861 | 0.166 | 3.08E-283 | 16 | 5.187 |
| Cxcl2 | 2.89E-215 | 2.474747537 | 0.614 | 0.095 | 9.32E-211 | 16 | 6.463 |
| AY036118 | 5.45E-52 | 1.07346048 | 1 | 0.964 | 1.76E-47 | 16 | 1.037 |
| Sfrp4 | 7.38E-17 | 0.504144997 | 0.4 | 0.205 | 2.38E-12 | 16 | 1.951 |
| Mt1 | 2.52E-16 | 0.243189124 | 0.864 | 0.637 | 8.15E-12 | 16 | 1.356 |
| Lpl | 1.32E-11 | 1.004176592 | 0.288 | 0.159 | 4.25E-07 | 16 | 1.811 |
| Tagln2 | 9.25E-10 | 0.650428706 | 0.515 | 0.406 | 2.99E-05 | 16 | 1.268 |
| Mt2 | 9.59E-06 | 0.145061123 | 0.502 | 0.365 | 0.309478173 | 16 | 1.375 |
